# Incidence and Risk Factors of Striae Distensae Following Breast Augmentation Surgery: A Cohort Study

**DOI:** 10.1371/journal.pone.0097493

**Published:** 2014-05-20

**Authors:** Denis Souto Valente, Rafaela Koehler Zanella, Leo Francisco Doncatto, Alexandre Vontobel Padoin

**Affiliations:** 1 Division of Plastic Surgery, Mãe de Deus Health System, Porto Alegre, Brazil; 2 Division of Dermatologic Surgery, Mãe de Deus Health System, Porto Alegre, Brazil; 3 ULBRA School of Medicine, Lutheran University of Brazil (ULBRA), Canoas, Rio Grande do Sul, Brazil; 4 Department of Medicine, Pontifical Catholic University of Rio Grande do Sul, Porto Alegre, Rio Grande do Sul, Brazil; University of North Carolina School of Medicine, United States of America

## Abstract

**Background:**

The significant increase in the popularity of breast augmentation surgeries has led to an increase in the number and types of complications; among these is the postoperative occurrence of Striae Distensae (SD). The objective of this study was to investigate the incidence of SD and describing its occurrence in association with age, breast implant volume, history of SD, history of pregnancies and breastfeeding, body mass index (BMI), changes in postoperative weight, smoking habits, and use of oral contraceptives.

**Methods:**

A cohort study was conducted and the patient data from a specific social group that underwent augmentation mammaplasty with silicone breast implants in a private clinic was analyzed.

**Results:**

563 patients entered the cohort, while 538 completed the study. The SD incidence was 7.06%. The risk was almost the double at 22–28 years of age and triple in women of 21 years of age or less. The women who did not use oral contraceptives were 2.59 times more likely of developing SD. A higher incidence of SD was observed among those with normal or low BMI values, smokers, and in those who had implants larger than 300 ml.

**Conclusions:**

Young age, larger implant volumes, smoking, and normal or low BMI values were the risk factors responsible for the development of SD; while using oral contraceptives was found to be a protective factor.

## Introduction

Augmentation mammaplasty is one of the most commonly performed aesthetic procedures [1]. This procedure aims to increase the breast volume in order to improve the patients' self-image and reduce their dissatisfaction with the size, shape, and appearance of their breasts. Silicone implants are most commonly used to achieve this objective [2]. The significant increase in the popularity of breast implant procedures in recent years has led to an increase in the number and types of complications associated with this procedure [3], [4]; among these complications is the postoperative occurrence of Striae Distensae (SD).

SD, also known as stretch marks or skin rupture occur when the tension applied to the skin is faster than its ability to expand. SD may be characterized by atrophic, linear, and parallel lesions. The lesions are usually running perpendicular to the Langer's lines, which represent the direction of minimum extensibility [5], [6].

The literature review performed using PUBMED and SciELO did not find any studies on the frequency of occurrence of SD following augmentation mammaplasty. We only found studies of reports or case series with SD after undergoing breast implant procedures and techniques for its treatment [3], [7], [8], [9], [10].

The main objective of this study was to investigate the occurrence of SD in women who had undergone breast augmentation surgery. Investigating the incidence of SD and describing its occurrence in association with age, skin color, breast implant volume, history of SD, history of pregnancies and breastfeeding, body mass index (BMI), changes in postoperative weight, smoking habits and use of oralcontraceptives.

## Methods

A cohort study was conducted and data of patients from a specific social group who underwent augmentation mammaplasty with silicone breast implants in a private clinic was analyzed. Data were collected from the patients' electronic records (RMD Clinic, RDTI Systems, Brazil) and digital image archives (Mirror PhotoFile, Canfield Scientific Inc., USA) from January 2005 to August 2012. The study protocol was approved by the Human Research Ethics Committee of the Lutheran University of Brazil (ULBRA), and is registered at the Brazilian Ministry of Health under the number 1111–1153–2184. This study did not affect the medical assistance provided to the patients because it was an analytical review of their medical records. All participants were made aware of the study and provided written consent before the surgery, so that their data, as well as before and after images, could be used in research. Written Consent Form was obtained from the guardians on behalf of the minors enrolled in this study. The individuals in this manuscript has given written informed consent (as outlined in PLOS consent form) to publish these case details.

The inclusion criteria were patients aged between 15 and 60 years who had undergone augmentation mammaplasty with placement of silicone implants and whom underwent a follow-up appointment that was performed at least 2 months after the breast augmentation surgery (i.e., postoperatively).

The exclusion criteria were concomitant mastopexy, incomplete medical records, absence of postoperative photos, use of corticosteroids, and previous breast surgery.

The early clinical signs of SD include itching, pain (in some cases), and flat and slight erythematous papular eruptions (Striae Rubra). SD are considered atrophic because of their characteristics; atrophy is a reduction in skin thickness as a result of a decrease in the number and volume of its elements; this translates into thinning, folding, drying, loss of elasticity, and hair rarefaction. Moreover, when the process of SD formation has already been established, the lesions have a whitish, almost pearly appearance (Striae Alba). SD can be characterized by the time they take to form–the newer aremore red, while the older are more white [5], [11]. Therefore, in this study, only red Striae were analyzed.

**Figure 1:**
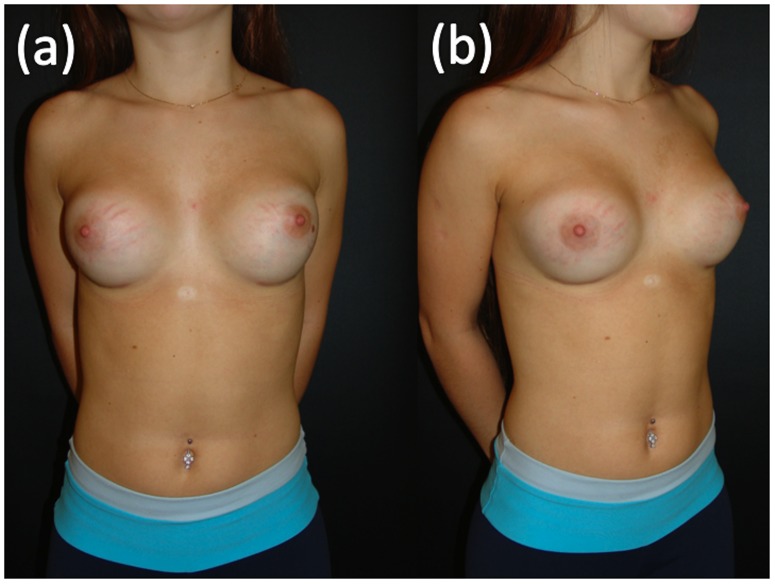
SD 3 weeks after 175 ml breast implants placement in a 16 years old woman. ‘(a)’ frontal view, ‘(b)’ oblique view.

**Figure 2:**
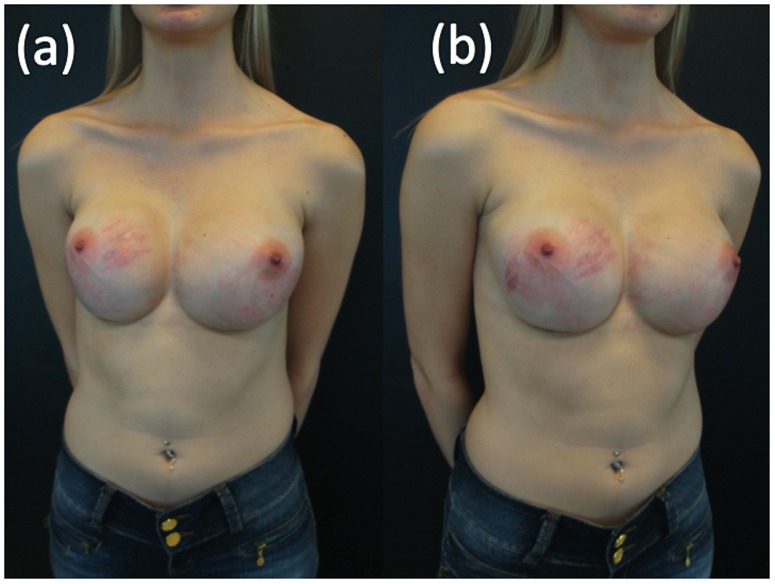
SD 6 weeks after 425 ml breast implants placement in a 24 years old woman. ‘(a)’ frontal view, ‘(b)’ oblique view.

Data regarding SD were collected from the electronic records in which information had been entered by the surgeon who performed the procedures. The criteria to define SD included the presence of cutaneous atrophy with acquired, reddish lesions of linear appearance and with a minimum width of 2 mm. Figure 1 and 2 show these findings. White or silver SD was considered old and was not accounted for by this study.

The presence of SD was associated with age, skin color, BMI, history of pregnancies and breastfeeding, smoking habits, use of oral contraceptives, changes in postoperative weight, and breast implant volume. These variables (except the changes in postoperative weight) were measured in the surgery day, and were also included in the electronic records.

Univariate and bivariate statistical analyses were performed using SPSS (IBM, USA) and Epi Info (CDC, USA), and the incidence ratio with its confidence intervals was used as the measure of effect. The variables that were considered included those showing statistical significance (p<0.1) using the likelihood ratio test (LRT). This level of significance was used in the analysis of known confounding factors to avoid the exclusion of important variables. Thus, some confidence intervals of 95% may include the unit. Categorical variables were included in the analysis as ordinals whenever the test for linear tendency was significant, without deviation from linearity.

**Figure 3:**
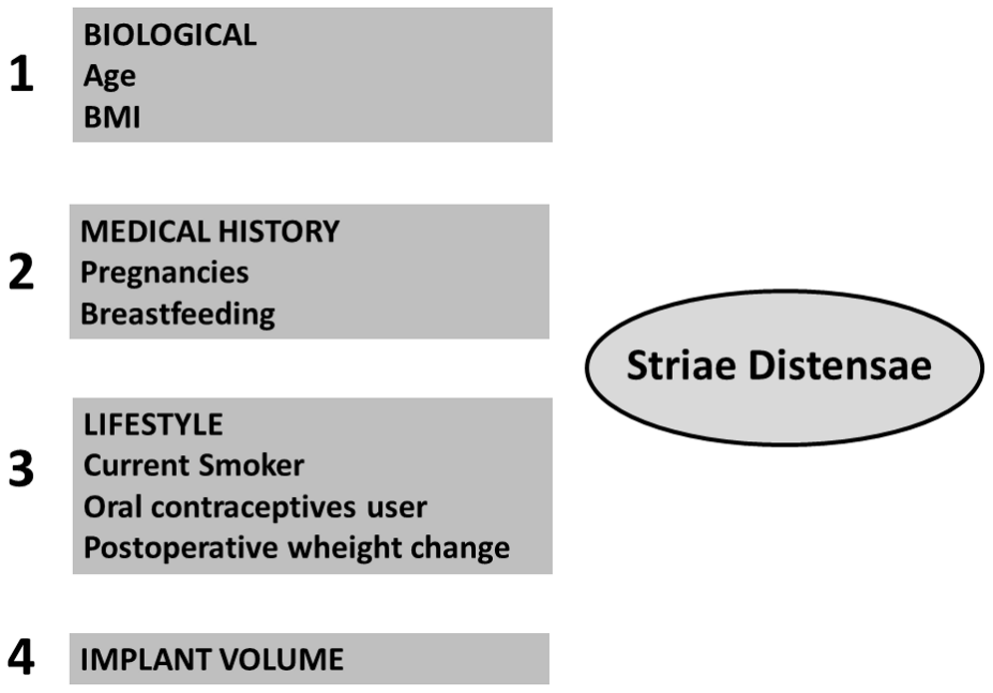
Hierarchical model of risk factors for the onset of SD.

Multivariate analysis was performed according to the hierarchical model defined a priori (Figure 3). In the first block (hierarchical level 1) all lifestyle habit variables were included, even those not considered to be significant by way of the bivariate analysis. The significant variables in this analysis were kept in the model and went into the adjustment of the next block, in this case those of the prior medical history (hierarchy level 2). Those with more than two categories remained in the model in a linear or categorical manner, depending on the best adjustment in the likelihood ratio test. For the following blocks of the model, the same procedure was performed. At the end of the above procedure, a final model of risk factors for the onset of SD was created. The selected variables in a given level remained in the subsequent models and were considered as risk factors for the onset of SD.

The statistical procedure utilized - logistic regression - expresses the results in terms of odds ratios, which are slightly larger (for risk factors) than the prevalence ratios, particularly because the outcomes studied are common. For protective factors, the odds ratio is less than the prevalence ratio. However, if there is no association between the duration of the SD and the risk factor, the prevalence odds ratio constitutes the best estimate for onset of SD [12].

From an ethics point of view, this study did not affect the medical assistance provided to the patients because it was an analytical review of their medical records. Medical confidentiality was assured because the person responsible for data collection, who had access to the patients' identities was only allowed to copy information regarding the variables included in this study.

## Results

In the period of January - 2005 to September – 2010, 563 women underwent the surgery. Among these, 21 women who did not undergo the follow up postoperative consultation after 2 months were excluded from the study. Of the remaining 542 women, 4 women did not have any data regarding SD in the medical records nor photos that permitted assessment; thus, they too were excluded from this study. Therefore, the sample included 538 women. The mean follow-up time was 8.3 months (range 2–41 months).In all patients the surgery performed was sub-glandular placement of texturized surface/round/silicone-filled devices.

Of the 538 women, 100% were Caucasian, 55.6% were aged between15 and 28 years, 57% had normal BMI, 32.4% had children, 15.1% had breast fed at least 1 child for more than2 months, 41.2% were active smokers, 77.7% had used oral contraceptives in the post operative period, and 42.9% had changes in postoperative weight of more than 5 kg.

The overall incidence of SD was 7.06%. This parameter was significantly higher in younger women; the risk of developing SD was almost double in women who underwent surgery at 22–28 years of age in comparison to those over 35 years of age. The risk of developing SD was triple in women who underwent surgery at the age of 21 years or less (Table 1).

**Table 1:** Incidence of SD and incidence ratio with confidence interval (CI) of the variables (N = 538).

	incidence (%)	Risk (CI)	N
**Years old (y.o.)**			
35 y.o. or more	4	1,00	122
29–35 y.o.	7	1,72 (0,98–3,01)	117
22–28 y.o.	7	1,78 (1,02–3,03)	134
15–21 y.o.	13	2,96 (1,84–4,76)	165
**BMI cut-offs (categories)**			
Over 24,9 (Overweight)	4	1,00	157
18,5–24,9 (Normal weight)	8	1,65 (1,20–2,25)	320
Under 18,5 (Underweight)	8	1,64 (1,11–2,51)	61
**History of pregnancies**			
No	7	1,00	364
Yes	6	0,88 (0,64–1,03)	174
**Breastfeeding history**			
No	7	1,00	457
Sim	6	0,92 (0,69–1,07)	81
**Current smoker**			
No	5	1,00	316
Yes	9	1,83 (1,44–2,32)	222
**Oral contraceptives use**			
Yes	5	1,00	418
No	12	2,59 (1,99–3,28)	120
**Postoperative weight change**			
Loss larger than 5 kg	6	1,00	92
Reduction of 5 kg or more	6	0,93 (0,61–1,39)	140
Gain of 5 kg	7	1,16 (0,80–1,66)	167
Gain larger than 5 kg	7	1,20 (0,83–1,75)	139
**Implant volume**			
150–225 ml	4	1,00	176
240–300 ml	6	1,73 (0,95–2,93)	184
310–375 ml	9	1,78 (1,04–3,03)	137
380–600 ml	13	2,71 (1,84–3,98)	41

The women who did not use oral contraceptives were 2.59 times more likely of developing SD as compared to those who did use oral contraceptives. With regard to BMI, a higher incidence of SD was observed among those with normal (18,5–24,9 kg/m2) and low (under 18,5 kg/m2) BMI values.

A linear association between the occurrence of SD and breast implant volume was observed, i.e., women who opted for implants larger than 300 ml had significantly more SD. Moreover, smokers were at a significant risk of developing SD.

There was no association between the occurrence of SD and pregnancy prior to the surgery, breastfeeding for more than 2 months, or changes in postoperative weight.

## Discussion

SD is considered to be dermal scars resulting from intrinsic stretching forces on weakened connective tissue. SD following breast augmentation is described as a rare complication [3], [4], [8], [9]. Just a few cases have been described, while there are probably many that exist. SD development can be disfiguring and devastating for the patient [4], [10].

Notably, in this study, the sample was obtained from a private clinic, corresponding to a specific social group that does not represent the general population. Therefore, the obtained results and their interpretation must take into account this specificity. The fact that 100% of the sample studied was Caucasian precludes extrapolation of the obtained results to the general population; however, it makes the external validity of this study higher for populations similar to the one used in the study.

The risk factors assessed for the development of SD have often been cited in the literature [5]. With the aim of investigating a potential association between the occurrence of SD and augmentation mammaplasty breast implant volume was included as one of the variables in this study. This variable was found to be associated with the occurrence of SD, because the risk of developing SD significantly increased when the implant volume was larger than 300 ml.

The physiopathology of SD remains unclear. The aetiological mechanisms proposed relate to hormones, and structural alterations to the integument. However, it may involve stretching of the skin, causing lesions in fibrilin microfibrils, which in younger women are likely to be more fragile and are therefore more susceptible to rupture [5], [11], [13], [14]. Rapid mechanical stretch produced by implant introduction seems to be the main factor leading to SD following breast augmentation [15].

Hormonal receptor expression is increased under certain conditions suggesting that regions undergoing greater mechanical stretching of the skin may express greater hormonal receptor activity. This fact may influence the metabolism of the extracellular matrix, causing SD formation. Alterations in hormone receptors occur within a well-defined period during the formation of SD. However, the functionality of hormone receptors varies during the different stages of SD development. Estrogen receptors doubled in skin with SD compared with healthy skin. The androgen and glucocorticoid receptors in the SD skin are also increased [16].

We observed a higher incidence of SD among women with normal or low BMI values. This may be due to the fact that women with higher BMI usually exhibit greater weight variation, causing sagging of the breast skin.

We did not find an association between the occurrence of SD and pregnancy prior to the surgery, breastfeeding for more than 2 months or changes in postoperative weight. We hypothesize that other variables could have been studied if the sample size had been larger, because the first two variables seemed to be protective factors, although there were no significant differences.

It is suggested that the subglandular implant allocation predisposes to SD more than the submuscular pocket because the muscle can reduce the skin stretching, acting as a type of splint^4^. Deformational changes such as SD potentially can occur with any implant placed into tissues that are tight and cannot accommodate it based on the width of the base and the soft tissue stretch of the breast. The greater size and projection of these implants potentially can have greater negative effects on the patients' tissues, including parenchymal thinning and the stretching of the skin [17]. In our study we did not study the implant location or the implant design.

Various complications related to silicone breast implants have been reported: capsular retraction with hardening of the breast, implant rupture, disappearance of the outer shell of the implant, calcification of the organic capsule, rupture with gel migration to neighboring tissues, postoperative dysmorphia, implant displacement, chronic pain, seroma, hematoma, infection, extrusion, changes in sensitivity, scarring problems, granuloma formation, skin inflammation with rashes, hives, migration to lymph nodes and peripheral nerves [18], [19], [20]. SD is another potential risk of breast augmentation that should be included routinely in informed consent documents because the onset of SD could give rise to legal action if the patient has not been informed of the risk.

The limitations of this study were the absence of data in the medical records regarding previous SD on breast skin or on other areas and difficulty in correlating the obtained results with those reported in the literature due to the lack of population studies on this subject (a 4.6% SD formation rate was previously described following breast augmentation) [15].

We utilized Confidence Intervals for being the most informative way of presenting the findings. The choice of the significance level of the test is completely arbitrary, in the majority of practical applications the value chosen as the significance probability *(p)* is 0.05 or 0.01; however there is nothing that formally justifies the use of these values in particular [21], [22]. In this study we used a value of p<0.1. Thus our manuscript assumes the probability of a 10% chance of committing a type I error; i.e. reject that there is no relationship between the onset of SD and the variables studied when this relationship does in fact exist. If our study is within this 10% of error, it would only mean that it was not able to (based on the available data), demonstrate the falsity that the onset of SD would be related to the studied variables; which completely differs from proving the veracity of this ratio [23].

## Conclusions

During the study period, the observed incidence of SD after augmentation mammaplasty with silicone implants was 7.06%. Young age, larger implant volumes, smoking, and normal or low BMI values were the riskfactors responsible for the development of SD, whereasusing oral contraceptives was found to be a protective factor.
